# Development of an Optical Calorimeter Sensor for the Arc Thermal Performance Value (ATPV) Determination on Arc-Rated Materials for Personal Protective Equipment

**DOI:** 10.3390/s26082352

**Published:** 2026-04-10

**Authors:** Fernanda Cristina Salvador Soares, Márcio Bottaro, Paulo Futoshi Obase, Rogério Masaro, Gleison Elias da Silva, Josemir Coelho Santos

**Affiliations:** 1 Divisão Científica de Tecnologia de Sistemas Elétricos, Instituto de Energia e Ambiente da Universidade de São Paulo, São Paulo 05508-010, Brazil; marcio@iee.usp.br (M.B.); obase@usp.br (P.F.O.); rogerio@iee.usp.br (R.M.); 2 Departamento de Engenharia de Energia e Automação Elétrica, Escola Politécnica da Universidade de São Paulo, São Paulo 05508-010, Brazil; gleisonelias@usp.br (G.E.d.S.); josemir.santos@usp.br (J.C.S.)

**Keywords:** optical sensor, arc rating, FBG sensor, ATPV, electric arc

## Abstract

**Highlights:**

**What are the main findings?**

- This study developed and tested an optical calorimeter sensor alongside traditional thermocouples to measure the arc-thermal-performance value (ATPV) of arc-rated fabrics;
- The results show that optical sensors, unaffected by electromagnetic interference and with faster response times, produce ATPV values lower than those measured with thermocouples, which could lead to different PPE classifications.

**What are the implications of the main finding?**

- The use of optical sensors challenges the accuracy of current standardized methodologies for determining arc ratings, suggesting that thermocouple-based measurements may overestimate protective performance;
- This difference has practical safety consequences, as misclassification of protective clothing could affect worker protection levels and influence compliance with safety regulations and standards.

**Abstract:**

The determination of the arc rating of arc-resistant materials for the manufacture of personal protective clothing is conducted by measuring the incident and transmitted energies through calorimetry using thermocouples coupled to copper discs during the electric arc events. In this study, custom calorimeters were constructed by incorporating both a thermocouple wire and an embedded optical-fiber temperature sensor, and the arc ratings of different fabrics were determined in terms of their arc-thermal-performance value (ATPV). The results revealed differences between the measurements obtained with the two sensor types. Notably, the absence of electromagnetic interferences generated by the arc current and the enhanced time response achieved with the optical-fiber temperature sensor signal led to an ATPV arc rating approximately 27% lower than that measured with the thermocouple. These findings underscore the importance of investigating the current methodology used for determining arc ratings to ensure accurate measurement of incident and transmitted energy.

## 1. Introduction

An arc-flash event entails a significant release of thermal energy through radiation, convection and heat conduction. It occurs when the insulation between live conductors deteriorates due to aging, surface tracking, treeing phenomena, and due to human errors during the maintenance procedures of energized electrical equipment [1,2,3]. Consequently, workers in the electrical sector exposed to this hazard may sustain burns, leading to severe injuries and, in some instances, fatalities. Therefore, these workers must utilize personal protective equipment (PPE), in addition to other collective and administrative protection measures. In the case of wearables, like clothing, balaclavas, and hoods, PPE is manufactured from natural or synthetic fabrics that incorporate specialized properties and technologies that are designed to provide arc-resistant performance.

The characterization of materials used in the manufacturing of PPE is achieved through assigning a numerical value to the product, which describes its protective performance when tested in accordance with an open-arc or a box test method. In the case of the open-arc method, this value is expressed either in kJ/m^2^ or cal/cm^2^, and it is referred to as the arc rating. The arc rating can be expressed by various quantities, such as the arc-thermal-performance value (ATPV), breakopen threshold energy (EBT50), or incident energy limit (ELIM).

According to the standard IEC 61482-1-1 [4], the ATPV represents the incident energy value at which the heat transferred through the test specimens is sufficient to meet the Stoll [5] criterion with a 50% probability. This value is calculated using a logistic regression analysis applied to data gathered during the test of a set comprising at least 20 samples. Conversely, the ATPV value assigned to a garment is any value equal to or less than the ATPV of the material from which it is constructed.

The methodology for determining this parameter is described in international standards such as IEC 61482-1-1 and ASTM F1959 [6]. The International Electrotechnical Commission (IEC) Standard, in method A, provides a comprehensive description of the setup for measuring incident and transmitted energy. This setup includes a supply bus, an arc controller, a recorder or data acquisition system, a pair of arc electrodes, and three panels with two internal sensors and two monitor sensors for each panel.

The incident energy is measured using calorimeters installed in the monitor sensors, while the energy transmitted by the material is measured using calorimeters installed in the panel sensors. These calorimeters are constructed from electrical-grade copper discs weighing 18 g, with a diameter of 40 mm and a thickness of 1.6 mm. Each calorimeter is equipped with a type K (NiCr–NiAl) thermocouple (TC), having a cross-sectional area of 0.05 mm^2^ (N° 30 AWG), but never larger, and a typical sensitivity of 41 µV/°C, which is inserted into a hole on the rear face at the center of the disc with a depth of 1.3 mm. The temperature rise over time is recorded during and after the application of the electric arc. Using the known thermophysical properties of copper, both incident and transmitted energies can be calculated.

To conduct the test, a minimum of seven electric arcs are initiated between the electrodes, with an alternating arc current of 8 kA ± 0.5 kA and an arc duration of at least 1.5 s from a power-frequency supply. This procedure is repeated to obtain a minimum of 20 samples for the subsequent statistical analysis. However, the high current involved in the application of the electric arcs results in significant electromagnetic interference (EMI) in the temperature signals of thermocouples within the calorimeters, leading to noise during the arc application. In practice, such noise is mitigated by employing an isolated channel acquisition system and by treating the signals with digital filters.

An alternative to minimize the influence of electromagnetic noise on signals is the use of temperature sensors based on fiber Bragg gratings (FBGs). In this type of sensor, a broad-spectrum light is injected into the fiber, and a narrow spectral component around the Bragg wavelength is reflected by the grating. As a result, this reflected spectral component is removed from the transmitted light. Most studies on this type of sensor are related to strain and temperature detection, with the strain response due to the physical elongation of the sensor with consequent changes in the grating spacing and changes in the fiber index due to photoelastic effects. The thermal response is due to the inherent thermal expansion of the fiber material and to the dependence of the refractive index on temperature [7]. Thus, sensors of this type have characteristics that allow for relating of the central wavelength (CWL) to the temperature to which the sensor is subjected. In addition to possessing interesting mechanical characteristics, such as small dimensions and mass, they also enable long-distance monitoring and signal multiplexing, as well as exhibit immunity to electromagnetic interference.

The use of optical-fiber temperature sensors in ATPV tests presents an alternative approach to avoid the EMI effects, eliminating the necessity for signal processing with digital filters and enabling the determination of the initial moment of temperature rise (t_0_). However, it is worth noting that, depending on the configuration, the response time of optical-fiber temperature sensors may be longer compared to thermocouples.

Although calorimeters employing optical-fiber temperature sensors have been investigated for several years [8,9], no reports were identified in the literature describing the use of an optical calorimeter for ATPV measurement. Hence, this study proposes a novel measurement system for determining arc ratings based on the development of a calorimeter equipped with both an optical-fiber temperature sensor and a thermocouple, enabling the comparison of the ATPV determinations for three different arc-resistant fabrics. A calorimetry setup was assembled, comprising a panel with two calorimeters for measuring transmitted energy and two external calorimeters to monitor incident energy during tests. This setup was constructed utilizing the facilities of the Laboratório de Ensaios de Vestimentas of the Instituto de Energia e Ambiente at the University of São Paulo, USP (LAEVe IEE/USP).

## 2. Materials and Methods

**Figure 1:**
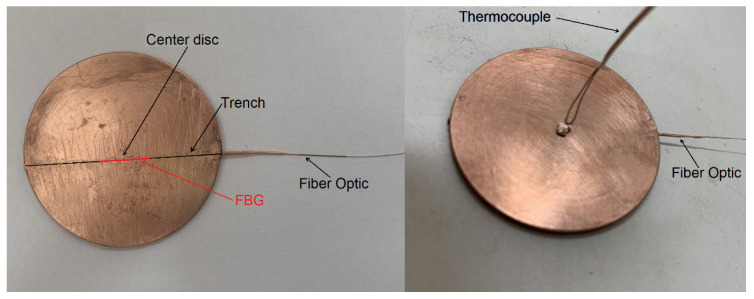
Calorimeter for determining ATPV with the attached thermocouple and an additional optical-fiber sensor embedded: front face (**left**) and back face (**right**).

Four calorimeters were constructed in compliance with the specifications of the standard IEC 61482-1-1. On the opposite side of the central hole, a V-groove was made in the disc using an ProtoMat C40 (LPKF Laser & Electronics, Garbsen, Germany) milling machine in order to create a trench 0.3 mm deep and 3 mm away from the diameter of the disc. Such a trench was used for placing the fiber-optic (FO) temperature sensor, which was composed of FBGs inscribed in a bare optical fiber with a clad diameter of 80 µm (main characteristics presented in Table 1). These FBGs were purchased from a commercial manufacturer (Proximion, Stockholm, Sweden). The FBG sensor was centered in the trench as indicated in Figure 1 (left). This assembly is hereafter called the optical-fiber temperature sensor (OFTS). The embedding process followed the technique detailed by Yulong Li et al. [10], wherein the fiber was initially coated with a thin copper plate using the electroless plating method, followed by an electroplating process. The optical fiber was then bonded to the copper disc using an electroplating process as well. Consequently, each calorimeter was equipped both with a thermocouple sensor and an FO temperature sensor, positioned at the same depth relative to the heat-receiving surface. In addition, a fifth calorimeter was constructed following the same procedure to replace one of the original calorimeters that was damaged during the ATPV determination.

As can be seen from the last row of Table 1, the average sensitivity of the FBG temperature sensor, after metallization, is 29.1 pm/°C.

The OFTS was linked to a Micron Optics optical interrogator, model SM230 (Roanoke, VA, USA), operating at a rate of 500 samples per second (S/s), while the thermocouples were connected to a thermocouple input module manufactured by National Instruments, model NI-9213 (Austin, TX, USA), with a sample rate of 100 S/s, adjusted via FPGA programming.

The relationship between the FBG central wavelength (CWL, in nm) and temperature, here referred to as calibration, was obtained using a Weiss Technik climatic chamber (Reiskirchen, Germany), model LabEvent LC/150/40/5, in conjunction with a digital thermometer used as a reference instrument. The digital thermometer comprised a Fluke Pt100 thermoresistance (Everett, WA, USA), model 5609, and a Presys digital indicator (São Paulo, Brazil), model ST-501-1. Calibration was conducted across temperatures ranging from 20 °C to 150 °C, in incremental steps of 10 °C. The calibration of the OFTS allowed for the values, measured in central wavelength (CWL), to be converted into temperature values, which were then compared with the measurements recorded by the thermocouples of the calorimeters (C1, C2, C3, and C4). During the ATPV testing of Fabric 1, the optical fiber of calorimeter C4 was damaged, so it was necessary to replace it with another calorimeter, which was designated as C5.

**Figure 2:**
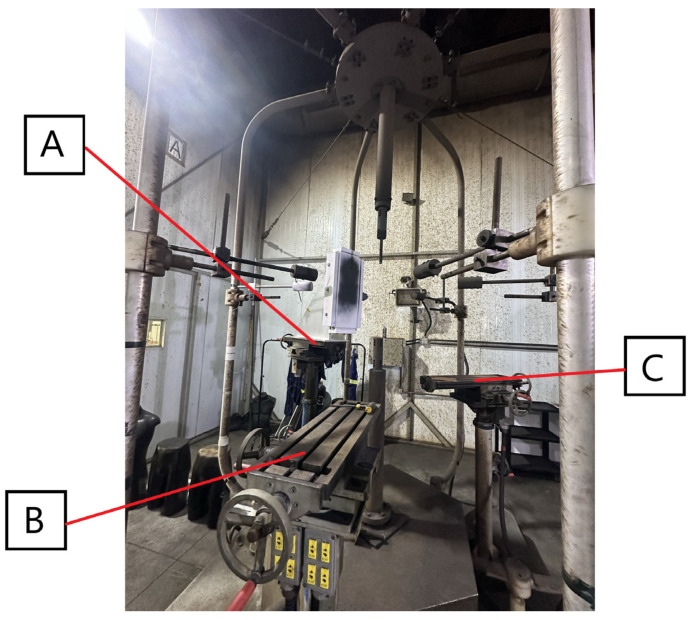
Placement of the panel and monitoring sensors at position A within the arc-flash testing cage at LAEVe IEE/USP.

**Figure 3:**
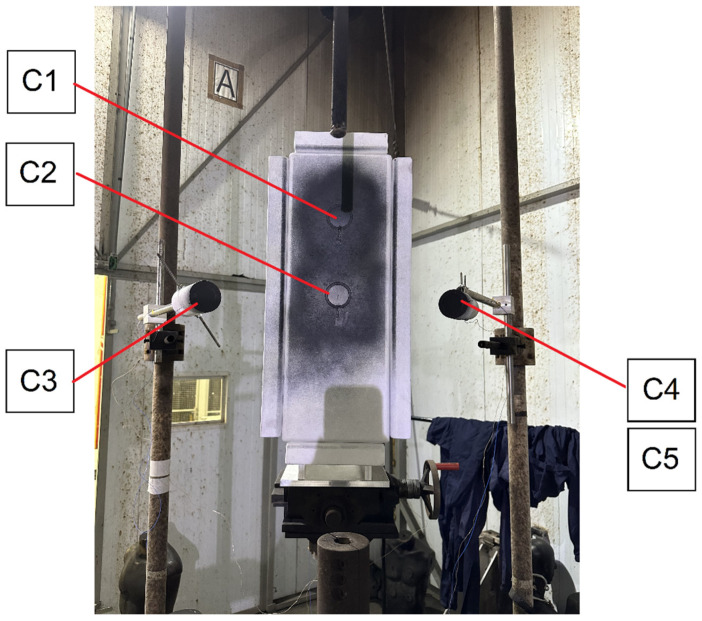
Test panel with attached calorimeters for measuring transmitted energy (C1 and C2) and monitor sensors (C3 and C4/C5).

Two calorimeters were mounted on the monitor sensors to measure incident energy, while the other two were attached to a panel to measure transmitted energy. Both the monitor sensors and panel board were fabricated from Marinite^®^ A (Athenas Brasil, Contagem, MG, Brazil) [11] and coated with black paint having an emissivity greater than 0.9. The assembly was placed in “position A” within the structural cage arrangement of the LAEVe IEE/USP, at a distance of 457 mm from the vertical electrodes. Subsequent to temperature measurements, the energies were computed and adjusted for the 305 mm distance using the inverse square law, as required by the reference standard. Positions B and C of the cage solely featured monitor sensors (refer to Figure 2). The calorimeters were labeled as C1 and C2 for measuring transmitted energy and C3 and C4/C5 for incident energy, as depicted in Figure 3.

To determine the incident and transmitted energies, temperature readings were recorded during and after the application of the electric arc. Utilizing the equations outlined in Standard IEC 61482-1-1, the transmitted energies (averaged from calorimeters C1 and C2) and incident energies (averaged from C3 and C4/C5 values) were calculated. Subsequently, the transmitted energy curve was then compared with the Stoll probability curve for second-degree burns. A value of “0” was recorded when the transmitted energy curve did not exceed the Stoll curve, while a value of “1” was assigned when it exceeded the Stoll curve at any time between 1 s after arc initiation (t_0_) and 30 s.

The ATPV determinations were conducted on the arc-rated (AR) fabric samples listed in Table 2. Each fabric specimen, measuring 700 mm × 350 mm, was placed individually on the test panel (Figure 3) to measure the energy transmitted through the material.

According to the reference standard, determining the ATPV of a material requires conducting a series of electric-arc shots on a minimum of 20 test specimens exposed to a range of incident energies. Typically, this is carried out using three sets of panels and monitor sensors, resulting in the minimum number of tests being achieved with seven electric arcs. However, in this study, since all measurements were made by a single panel and two monitor sensors, it was necessary to apply 22 electric arcs for the Fabric 1 and 20 electric arcs for the Fabrics 2 and 3. These electric arcs had incident energies ranging according to the test criteria outlined in IEC 61482-1-1. The electric arcs were generated with an alternating current of 8 kA ± 0.5 kA, with arc durations between 0.08 s and 0.32 s, i.e., 5 to 19 mains cycles at 60 Hz.

Following a comparison of the transmitted energy with a Stoll curve across the electric arcs, a logistic regression analysis was applied utilizing a Microsoft Excel^®^ add-in that extends the standard statistical features of Excel, called Real Statistics Resource Pack [12]. Subsequently, the ATPV determined utilizing the fiber-optic calorimeter could be compared with the ATPV obtained with the calorimeter equipped with a standardized thermocouple sensor.

## 3. Results

**Figure 4:**
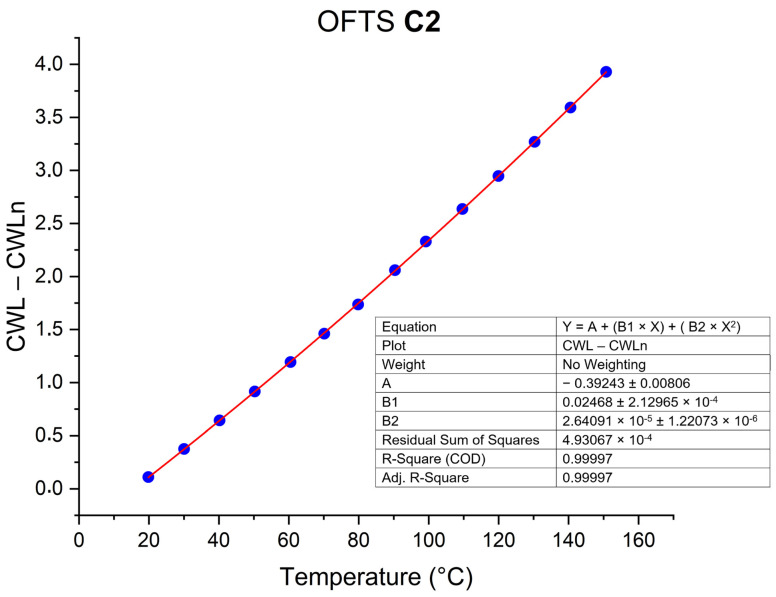
Example of the calibration procedure applied to OFTS. Polynomial fitting curve for OFTS C2.

Generally, in the calibration procedure, temperature is physically the controlled variable used to generate different wavelength values and model the sensor response; therefore, it could naturally be placed on the x-axis. Since, within the temperature range of interest, the sensor response is not strictly linear, it is better described by a polynomial model, as in the example shown in Figure 4. Following approaches commonly adopted for fiber-optic temperature-sensing calibration [13,14], after a proper data-processing, wavelength is treated as the independent variable (x-axis), enabling the direct formulation of a second-degree polynomial equation that converts wavelength into temperature, which corresponds to the practical objective of the measurement process.

The calibration results of the OFTS were obtained through a second-degree polynomial fit using the Origin^®^ software (Origin 2022, 64-bit SR-1, 9.9.0.225 Academic), with the corresponding coefficients shown in Table 3.

Consequently, the values measured in CWL can be translated into temperature values and then compared with the measurements recorded by the thermocouples of calorimeters for each of the electric arcs applied.

**Figure 5:**
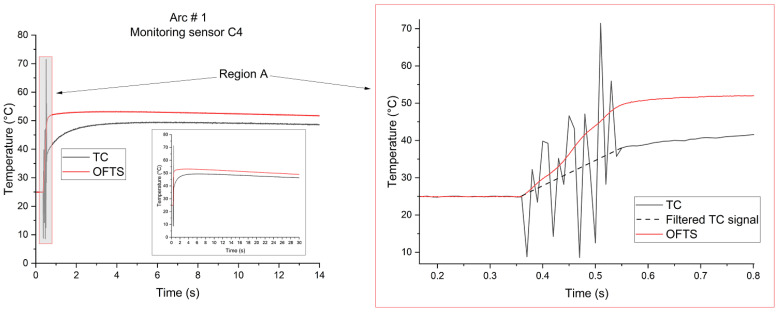
Temperature measurement of the monitor sensor (calorimeter C4) with both the thermocouple and OFTS in electric arc number 1, during the ATPV determination of Fabric 1.

Figure 5 shows the temperature signals recorded by the monitor sensor C4 during the application of electric arc 1 (10 mains cycles) in the ATPV determination for Fabric 1. The thermocouple signal, depicted by the solid line (TC), is not filtered and is shown to demonstrated the amplitude of the noise. The dashed line represents the TC signal after application of the digital filter. Immediately after the extinction of the electric arc, it was observed that the maximum temperature measured by the fiber-optic sensor is approximately 10 °C higher than that recorded by the thermocouple sensor. The OFTS reached its maximum temperature approximately 3 s after the arc event, whereas the thermocouple recorded its maximum temperature only after 5.5 s from the arc application. Given that the OFTS presents a faster thermal response, it may follow the temperature rise more quickly immediately after arc exposure. In contrast, considering that the thermocouple presents higher thermal inertia and a slower response time, when it approaches its peak response, the calorimeter has already started cooling due to the interruption of the heat source (electric arc), preventing the thermocouple from registering the same maximum temperature registered by the OFTS. As shown in the inset of Figure 5, after 30 s, when the temperature signal recorder was stopped, the temperature difference between the sensors decreased to 2.7 °C. This behavior was consistently observed in all performed measurements. By zooming in upon Region A of Figure 5, it becomes apparent that the rate of temperature rise in the optical-fiber sensor is faster than that of the thermocouple center. Additionally, it is worth noting that the thermocouple signal exhibits electromagnetic noise, which is not observed in the OFTS. In the first edition of Standard IEC 61482-1-1, since the determination of the initial temperature (T_0_) relied on the onset of the temperature signal rise, the electromagnetic noise in the thermocouple presented a challenge that required treatment with digital filters. In the second edition of the Standard, although the arc-initiation time aligns with the onset of the electric-arc current signal, the use of digital filters to remove noise is recommended. However, it is essential that these filters maintain the waveform integrity and do not introduce a time-shift behavior. Consequently, the use of temperature sensors immune to EMI could obviate the need for synchronized acquisition of temperature and arc-current signals, as well as the need for digital filters.

All sensor temperature responses were converted to heat energy (both incident and transmitted energies), while considering the temperature dependence of the heat capacity of copper, using Equation (1) below, as defined in IEC 61482-1-1 Standard. Table 4 presents the number of mains cycles used in each of the electric arc and the incident energies calculated from the temperature records.

(1) $$Q_{i}=\frac{10\times m_{i}}{a_{i}\times 63,546}\times \left[A\times \left(T_{i}-T_{0}\right)+B\times \left(\frac{{T_{i}}^{2}-{T_{0,i}}^{2}}{2}\right)+C\times \left(\frac{{T_{i}}^{3}-{T_{0,i}}^{3}}{3}\right)+D\times \left(\frac{{T_{i}}^{4}-{T_{0,i}}^{4}}{4}\right)+E\times \left(\frac{1}{T_{0,i}}-\frac{1}{T_{i}}\right)\right],$$

where

Qi is the heat energy for sensor i in kJ/m^2^;
A = 17.72891;
B = 28.09870 × 10^−3^;
C = −31.25289 × 10^−6^;
D = 13.97243 × 10^−9^;
E = 0.068611 × 10^−6^;
$m_{i}$ is the mass of the copper disc of sensor i in g;
$T_{i}$ is the temperature of the copper disc of sensor i at each sampling time in K;
$T_{0,i}$ is the initial temperature of the copper disc of sensor i at t_0_ (arc initiation time) in K;
$a_{i}$ is the area of the exposed copper disc of sensor i in cm^2^

**Table 4:** Incident energies measured with both the thermocouple sensor (TC) and the optical-fiber temperature sensor (OFTS), along with the results of the comparison of the panel sensor response with Stoll curves. The highlighted results (yellow brackground color) show the differences in the evaluation criteria of the Stoll curves (burn/no-burn).

Arc #	Fabric 1				Fabric 2				Fabric 3			
	TC		OFTS		TC		OFTS		TC		OFTS	
	Ei (cal/cm^2^)	Stoll	Ei (cal/cm^2^)	Stoll	Ei (cal/cm^2^)	Stoll	Ei (cal/cm^2^)	Stoll	Ei (cal/cm^2^)	Stoll	Ei (cal/cm^2^)	Stoll
1	6.27	0	6.50	1	8.82	1	9.65	1	5.32	0	5.61	1
2	7.42	1	7.98	1	8.91	1	9.75	1	7.28	1	7.66	1
3	6.38	0	6.79	1	5.46	0	5.89	0	7.85	1	8.32	1
4	6.41	0	6.67	0	5.33	0	5.77	0	6.32	1	6.49	1
5	5.03	0	5.25	0	5.70	1	6.28	1	4.57	0	4.77	0
6	6.85	0	6.11	0	7.21	1	7.86	1	5.45	0	5.75	0
7	6.19	0	4.89	1	5.37	0	5.73	0	4.28	0	4.49	0
8	7.23	0	6.78	0	5.50	0	5.94	1	5.63	1	5.99	1
9	6.71	0	6.14	1	6.10	0	6.72	1	3.78	0	3.90	0
10	8.68	1	7.67	1	5.06	0	5.38	0	4.84	0	5.07	0
11	8.61	1	5.30	1	4.78	0	5.07	0	6.49	1	6.77	1
12	7.11	1	6.45	1	3.42	0	3.63	0	5.12	0	5.43	0
13	7.23	0	5.71	1	4.69	0	5.01	0	4.94	0	5.15	0
14	6.89	0	5.03	0	6.19	0	6.73	1	6.46	0	6.87	0
15	10.06	0	8.15	0	6.22	1	6.71	1	6.51	1	6.92	1
16	10.52	1	10.27	1	6.59	1	6.94	1	8.71	1	9.33	1
17	10.75	1	10.18	1	6.43	1	6.77	1	7.11	0	7.57	1
18	9.78	1	9.32	1	5.55	1	5.80	1	6.66	0	6.95	1
19	11.67	1	11.89	1	4.88	0	5.14	1	5.04	0	5.41	0
20	3.16	0	2.59	0	6.90	1	7.36	1	6.09	0	6.24	0
21	3.15	0	2.41	0	-		-		-		-	
22	3.07	0	3.30	0	-		-		-		-	

During Fabric 1 data acquisition, in arc number 6, the optical fiber of the calorimeter C4 was struck by material expelled by the electric arc and, consequently, was ruptured. As a result, the incident energy values from the optical-fiber sensor from arcs 6 to 22 refer solely to the temperature values recorded by the calorimeter C3.

Table 4 also provides the results of comparing the energy transmitted from each electric arc with the Stoll curve (Stoll criterion column). It can be noted that for arcs 1, 3, 7, 9, and 13, discrepancies were observed in the assessment of the probability of second-degree burns. The results that showed differences in the evaluation criterion of the Stoll curve (burn/no-burn) are highlighted in Table 4.

**Figure 6:**
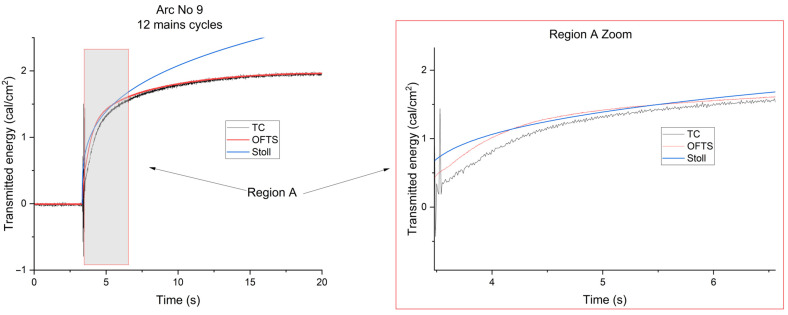
Energies transmitted by the fabric under evaluation following the application of arc no. 9, as measured both by the thermocouple sensor (TC) and an optical-fiber temperature sensor (OFTS), along with a comparison via the Stoll curve.

As illustrated in Figure 6, which depicts the transmitted energy curves measured by the thermocouple sensors and the optical fiber, along with the standard Stoll curve during the application of arc no. 9, it is evident that the transmitted energy curve measured with the OFTS reached the Stoll curve, whereas this alignment did not occur with the curve recorded by the thermocouple sensor. This discrepancy can be attributed to the faster response time of the OFTS compared to the thermocouple.

**Figure 7:**
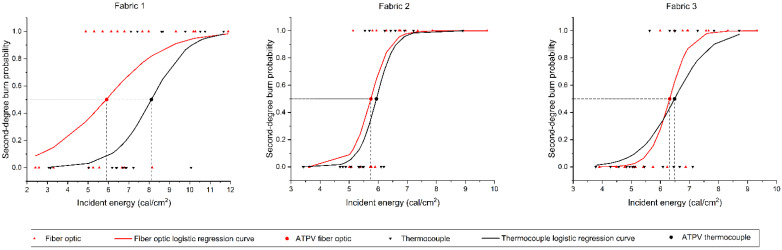
Logistic regression curves based on the evaluation of the probability of second-degree burns from measurements with both a thermocouple sensor (TC) and an optical-fiber temperature sensor (OFTS).

Table 5 summarizes the ATPV values obtained for the three fabrics using both the thermocouple and OFTS. The results show that Fabric 1 exhibited the largest discrepancy between the two measurement techniques, with the ATPV determined by the OFTS being approximately 27% lower than that obtained with the thermocouple (5.9 cal/cm^2^ versus 8.1 cal/cm^2^), as illustrated in Figure 7. This reduction exceeds the typical measurement uncertainty reported in the literature [15], highlighting the influence of the sensor response time and electromagnetic interference on the thermocouple readings. In contrast, Fabrics 2 and 3 showed minimal variation between the two measurement methods, with differences of only 3%, which fall within the expected uncertainty range, reported to be about 10% within this energy range [15]. Overall, the results confirm that while the OFTS may yield lower ATPV values under certain conditions, it provides a more accurate and interference-free measurement of the true thermal energy transmitted through the protective fabric.

It is evident in Figure 7 that, compared to Fabric 1, Fabrics 2 and 3 exhibited a smaller mixed-zone (“S-shaped” function of the logistic model), and this can be an important indicator of fabric performance and test uncertainty.

Variations in ATPV determination are a current challenge for textile manufacturers and laboratories, a challenge that can be mitigated through the use of measurement technologies offering higher accuracy and electromagnetic immunity [16,17].

**Figure 8:**
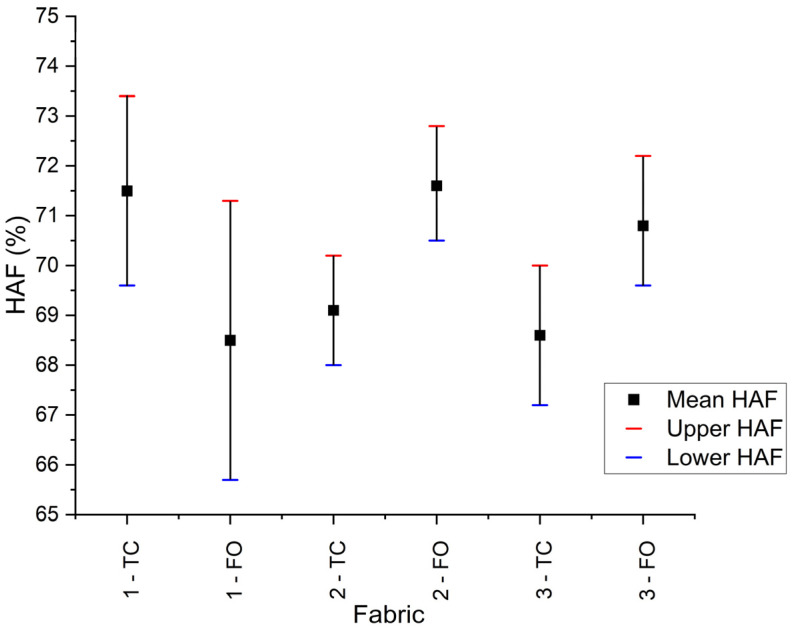
HAF results for Fabric 1, Fabric 2, and Fabric 3 measured with the thermocouple and OFTS.

Finally, for comparative purposes, the heat attenuation factor (HAF) of the fabric under evaluation was also determined using both types of sensors, despite the second edition of the IEC 61482-1-1 standard no longer mandating this measurement. The HAF represents the percentage of incident energy attenuated by a material at an incident energy level corresponding to the ATPV, and its determination was required in the first edition of the IEC 61482-1-1 standard [18]. Figure 8 presents the HAF results for Fabrics 1 to 3. Fabric 1 exhibited the widest confidence interval among the fabrics, indicating that the heat attenuation factor is more susceptible to variation with energy. The values obtained using both types of calorimeters are consistent within the reported confidence levels.

## 4. Discussion

Compared to the thermocouple sensor, the measurements obtained with the OFTS exhibited faster response times and immunity to electromagnetic interference (EMI), which are two major limitations of the existing calorimetric setup described in the IEC 61482-1-1 and ASTM F1959 standards. Faster response times with the OFTS were observed for all of the measurements and fabrics.

It was also noted that comparing the average temperature rise transmitted through the fabrics to the internal calorimeters revealed discrepancies in the comparison with the Stoll curve in the same incident energy from the same electric arc. As a result, Fabric 1’s arc rating, in terms of ATPV, calculated for measurements made with the OFTS was approximately 27% lower than the value obtained through measurements made with the thermocouple.

The discrepancy between the OFTS’s and thermocouple sensor’s readings was less pronounced for Fabrics 2 and 3, with differences within the typical uncertainty range. This indicates that fabric construction and heat transfer characteristics influence the impact of the sensor dynamics on the ATPV outcomes.

Although the study used a single-sensor set per test instead of three, as required by the standards, the improvement in measurements with the OFTS suggests a strong potential for standardization. Further work should evaluate the repeatability of the OFTS measurements across different ATPV ranges, as well as the long-term stability of the metal-coated optical-fiber sensor under repeated arc exposure.

## 5. Conclusions

The results obtained in this study demonstrate that the calorimeter equipped with an optical-fiber temperature sensor (OFTS) represents a significant improvement in the measurement of incident and transmitted energies during ATPV tests. The faster thermal response of the OFTS allows it to detect the transient heating process with higher fidelity, in all cases, capturing the real peak of transmitted energy, which might be partially underestimated by thermocouples due to thermal inertia.

Additionally, since the sensitivity of the measurements for different types of fabric proved to be dependent on the type of sensor, in all fabric cases, with a larger mixed zone, the sensitivity of the measurements made with the FO sensor was greater, suggesting that the sensor response characteristics become even more critical for materials exhibiting complex transient heat propagation or nonuniform structures. Due to the variation in the method, recently explored by manufacturers and laboratories, the contribution of a more accurate measuring systems can be crucial for mitigating weaknesses in the process and improving the accuracy of the results.

The findings highlight potential implications for PPE certification. Since the ATPV value defines the arc-rating classification, any systematic overestimation—such as that observed with thermocouples—may lead to incorrect labeling of protective clothing. This misclassification could compromise worker safety by giving a false sense of protection under high thermal stress conditions.

Furthermore, the present study underscored the significance of scrutinizing the methodology employed to ascertain arc rating. Ensuring accurate measurement of incident and transmitted energy is paramount to the safety of workers who rely on PPE. Enhanced accuracy in these measurements can also affect incident energy studies, including empirical estimates such as those outlined in IEEE 1584 [1].

This article is a revised and expanded version of a paper entitled “Determinação do ATPV utilizando calorímetro baseado em sensor óptico”, which was presented at “IEEE ESW-Brasil 2023, São Paulo—SP, Brazil, 2023 [19].

## Figures and Tables

**Table 1:** Main characteristics of the fiber-optic temperature sensor.

	FBG				
Characteristic	C1	C2	C3	C4	C5
Length (mm)	10	10	10	10	10
Central wavelength (nm)	1540.2	1540.2	1559.9	1559.9	1569.7
Bandwidth (nm)	0.28	0.27	0.27	0.27	0.28
Reflectivity (%)	99	90	90	97	99
Sensitivity after Metallization (pm/°C)	28.4	29.1	28.8	29.6	29.8

**Table 2:** Main characteristics of the arc-rated fabrics evaluated in this study.

Fabric	#1	#2	#3
Description	100% cotton fabric	88% cotton/12% nylon knitted fabric	88% cotton/12% nylon fabric
Spec weight (g/m^2^)	290	220	260
Color	Navy blue	Beige	Sky blue

**Table 3:** Polynomial fit parameters for calibrating fiber-optic sensors: T = A + (B1 × (CWL − CWLn)) + (B2 × ((CWL − CWLn)^2^)).

Parameter	C1	C2	C3	C4	C5
A	−5.70775	16.27966	26.73297	92.15766	−3.593
B1	40.11772	38.09871	34.0857	33.93258	40.363
B2	−1.24238	−0.98746	−0.80449	−0.72326	−1.253
CWLn	1541	1540	1560	1560	1569

**Table 5:** ATPV results for Fabric 1, Fabric 2, and Fabric 3 measured with the thermocouple (TC) and optical-fiber temperature sensor (OFTS).

Fabric	ATPV (cal/cm^2^) with TC	ATPV (cal/cm^2^) with OFTS
1	8.1	5.9
2	5.9	5.7
3	6.5	6.3

## Data Availability

The experimental data that supports the findings of this study are available by contacting the corresponding author, F.C.S.S., upon reasonable request.

## Abbreviations

The following abbreviations are used in this manuscript:

ATPV	Arc-Thermal-Performance Value
CWL	Central Wavelength
EBT	Breakopen Threshold Energy
ELIM	Incident Energy Limit
EMI	Electromagnetic Interference
FO	Fiber Optic
FBG	Fiber Bragg Grating
IEC	International Electrotechnical Commission
OFTS	Optical-Fiber Temperature Sensor
PPE	Personal Protective Equipment
TC	Thermocouple
